# Spectrum adequacy of antibiotic regimens for secondary peritonitis: a retrospective analysis in intermediate and intensive care unit patients

**DOI:** 10.1186/s12941-015-0110-4

**Published:** 2015-11-05

**Authors:** Cathérine L. Steinbach, Christoph Töpper, Thomas Adam, Martin G. Kees

**Affiliations:** Department of Anesthesiology and Intensive Care, Charité Universitätsmedizin Berlin—Campus Benjamin Franklin, Hindenburgdamm 30, 12200 Berlin, Germany; Labor Berlin GmbH, Department of Microbiology, Clinical Consulting, Sylter Str. 2, 13353 Berlin, Germany

**Keywords:** Abdominal infection, Antimicrobial management, Secondary peritonitis, Nosocomial infection, Surgery

## Abstract

**Background:**

Secondary peritonitis requires surgical source control and adequate antimicrobial treatment. Antimicrobial regimens are usually selected according to local susceptibility data of individual pathogens against single agents, but this neglects both the polymicrobial nature of the infection and the use of combination therapy. We analysed the probability of common regimens to cover all relevant pathogens isolated in one patient (“spectrum adequacy rate”, SAR) in a real-life data set.

**Methods:**

Data from 242 patients with secondary peritonitis (88 community acquired, 154 postoperative cases) treated in our IMCU/ICU were obtained retrospectively. The relative frequency of pathogens, resistance rates and the SAR were analysed using the free software R.

**Results:**

Enterococci were isolated in 47.1 % of all patients, followed by *Escherichia coli* (42.6 %), other enterobacteriaceae (33.1 %), anaerobes (29.8 %) and *Candida* spp. (28.9 %). Resistance patterns were consistent with general surveillance data from our hospital. The susceptibility rates and SAR were lower in postoperative than in community acquired cases. The following regimens yielded a SAR > 95 % when enterobacteriaceae only were considered: piperacillin/tazobactam + gentamicin, cefotaxim (only for community acquired cases), cefotaxim + gentamicin, meropenem, tigecycline + gentamicin or tigecycline + ciprofloxaxin. When enterococci were also considered, all betalactam based regimens required combination with vancomycin or linezolid for a SAR > 95 %, whereas TGC based regimens were not compromised. As for *Candida* spp., the SAR of fluconazole was 81.9–87.5 %.

**Conclusions:**

This study demonstrates a rational approach to assess the adequacy of antimicrobial regimens in secondary peritonitis, which may help to adjust local guidelines or to select candidate regimens for clinical studies.

## Background

Secondary peritonitis (due to a gastrointestinal perforation or leakage) is among the leading causes of community acquired sepsis. Equally, postoperative secondary peritonitis is a dreaded complication of intestinal surgery with a high burden of morbidity and mortality. It is easy to understand that these are typically polymicrobial infections. Second to surgical source control (i.e. closure of the perforation and lavage), antimicrobial therapy has an important role. Inadequate antimicrobial therapy—too late, too little, or wrong spectrum—has been shown to affect the clinical evolution and outcome [1–3].

This link is less direct than e.g. in pulmonary infections where surgery has no role and antimicrobial therapy is the only causative therapy. Favourable outcome may also be achieved with limited spectrum therapy, directed only against the most common and pathogenic isolates (aerobic Gram-negative bacilli and anaerobes). In severe cases, however, the presence of drug-resistant organisms and also isolates of questionable or facultative pathogenicity (such as *enterococci* and yeasts) should be considered, and coverage of all isolated pathogens is preferable.

Ideally, recommendations for empiric treatment should be custom tailored to the specific population, so that initial treatment will offer an adequate spectrum in most and an excessive spectrum in few cases. Whereas it is reasonable to consider the relative frequency of isolated species and their individual resistance rates, this approach neglects the polymicrobial nature of secondary peritonitis. Co-infection by two or more pathogens with different resistance patterns will lead to a higher percentage of inadequate treatment than individual resistance rates suggest. (For instance, the susceptibility rates of *Escherichia coli* to cephalosporins are notably higher than to fluoroquinolones, whereas the opposite is the case for many other enterobacteriaceae, e.g. *Enterobacter* spp. or *K. pneumoniae* [4].) The simultaneous evaluation of all pathogens identified in one patient for susceptibility against an antimicrobial regimen seems a more logical approach. To use an analogy to a famous pad-and-pencil game: the battleship is only sunk when all parts of it have been hit.

With the present study, we provide a retrospective analysis of the spectrum adequacy of selected antimicrobial regimens in a real-life data set of pathogens cultured in patients with secondary peritonitis who were admitted to the surgical intermediate and intensive care unit (IMCU/ICU).

## Methods

### Patient selection and data acquisition

Using our hospital-wide electronic patient file management system, we screened every patient who had been discharged from (or had deceased in) our IMCU/ICU between 1 August 2012 and 31 January 2014 for the presence of positive intraabdominal culture results. On the basis of the discharge letter and surgical reports, all patients who had undergone surgery for a confirmed diagnosis of secondary peritonitis were selected, excluding patients who had not undergone surgery, other forms of peritonitis, samples obtained during elective surgery without evidence of infection, or misspecified samples from other sources. Patients who had been newly admitted for abdominal symptoms, and in whom the intestinal perforation was unrelated to previous surgery (e.g. perforation of a gastric ulcer or of the sigmoid colon), were classified as community-acquired cases (c.a.). When peritonitis was a complication of recent surgery (e.g. anastomotic leak), it was classified as postoperative secondary peritonitis (p.op.). For each patient, only microbiological results from the first laparotomy were included, not from subsequent relaparotomies. If a patient had valid swab results for community acquired and subsequently for postoperative secondary peritonitis, only the first (community acquired) episode was considered. The anatomical site of the lesion was registered. Anatomically well defined lesions were categorised as stomach/duodenum, small intestine or colon. Otherwise, e.g. in cases of multiple lesions or in intestinal ischemia, it was categorised as “other”. In addition to microbiological data, age, sex, length of stay in the IMCU/ICU and in the hospital after admission on IMCU/ICU, and death was obtained. No data on the individual antimicrobial treatment could be collected, since these informations are not recorded in the electronic patient file management system. For elective intestinal surgery, the standard for perioperative prophylaxis is cefuroxime 1.5 g, in the case of colonic surgery in combination with metronidazole 500 mg, which is repeated in cases of prolonged surgery, but is not extended beyond the end of the procedure. For laparotomy for suspected intestinal perforation, most patients in our institution receive either the same regimen as for elective surgery (cefuroxime ± metronidazole) or piperacillin/tazobactam 4.5 g preoperatively, and treatment is adjusted subsequently according to the intraoperative findings and patient specific risk factors.

The responsible ethics committee of the Charité Universitätsmedizin Berlin gave approval for publication of the study results (reference number EA2/045/15).

### Microbiological diagnostics

In our hospital, swabs are preferred over other sampling techniques (e.g. inoculation of peritoneal fluid into blood culture bottles [5]), but results were considered for this analysis independent of the used material as long as they originated from the peritoneal cavity. Routinely, a swab (eSwab™, BD, Heidelberg, Germany) is taken from the interenteric fluid immediately after opening of the peritoneal cavity; further samples may be obtained from suspect sites at the discretion of the surgeon. Samples are transferred to the central microbiological laboratory (Labor Berlin GmbH) and processed using standard techniques: swabs were applied onto routine microbiological media (Columbia blood, chocolate, McConkey, Schaedler, Sabouraud agar plates and thioglycolate broth), incubated at 36 °C with or without CO_2_, under aerobic or anaerobic conditions, respectively. Aerobic media were read on day 1 and 2, anaerobic media on day 2. Aerobic bacteria were identified by biochemical methods (Vitek2, bioMérieux, France) or by mass spectrometry (microflex with Biotyper software, Bruker Daltonics, Germany, or VitekMS, bioMérieux, France). Vitek2 was used for antimicrobial testing. For selected microorganisms we used standard agar diffusion procedures (Kirby and Bauer) or E-Test (bioMérieux, France) to establish antibiograms [6, 7]. Interpretation of breakpoints was done using EUCAST clinical breakpoint tables (http://www.eucast.org/clinical_breakpoints/). Divergent from the EUCAST expert rules in antimicrobil susceptibility testing [8], the susceptibility of ESBL producing enterobacteriaceae against betalactam/betalactamase inhibitor combinations (SAM, TZP) and cephalosporins was reported as resistant (instead of as tested with a warning on uncertain therapeutic outcome).

### Data analysis

The statistical package ‘R’ (V3.1.1 for MacOSX, R foundation for statistical computing, Vienna, Austria) with ‘RStudio’ (RStudio Inc., Boston, MA, USA) was used for the analysis, Prism (V6.0d for MacOSX, GraphPad Software, La Jolla, CA, USA) for graphical presentation of the results.

The following tasks were performed by an R-script:

1. Elimination of copy strains.
2. Classification of isolates into the following categories: *E. coli*, enterobacteriaceae other than *E. coli*, non-fermenting Gram-negative rods, *enterococci*, *streptococci*, *S. aureus*, coagulase-negative *staphylococci*, anaerobes, *Candida* spp., and others.
3. Tabulation of resistance/susceptibility for each of these categories.
4. Tabulation of the prevalence of the pathogen categories sorted by diagnosis and anatomical location of the lesion.
5. Calculation of the spectrum adequacy rate (SAR) for selected antimicrobial regimens. The spectrum was adequate if all isolated enterobacteriaceae or all enterobacteriaceae plus *enterococci* in the same patient were susceptible to the tested regimen; *candida* spp. were tested separately. The SAR is reported as percentage of the patients within the same setting (community acquired or postoperative).

The results of 3 and 4 were used to describe and compare the results with local susceptibility data or other studies. Task 5 was the primary purpose of this study.

## Results

**Fig. 1:**
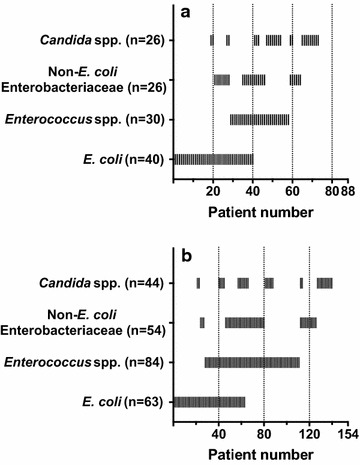
Concomitant isolation of selected pathogens in single patients with community acquired (**a**) or postoperative (**b**) secondary peritonitis

**Fig. 2:**
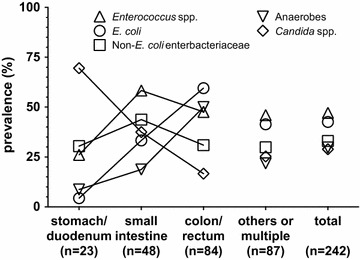
Prevalence of selected groups of pathogens isolated in patients with secondary peritonitis, according to site of lesion

The relevant population was composed of 242 patients who had undergone surgery for confirmed secondary peritonitis, among which 88 had community acquired and 154 postoperative peritonitis. Case characteristics are displayed in Table 1. A total of 654 different strains was isolated, details are shown in Table 2. The number of isolated pathogens per patient was equal in community acquired and postoperative disease (median 2, interquartile range 1–4), the maximum number observed being eight. Figure 1 gives a graphical representation of the simultaneous isolation of relevant pathogens. Overall, *Enterococcus* spp. was the most prevalent category of pathogens, and was isolated in a high percentage of cases independently of the clinical setting or the anatomical site of intestinal lesion (Fig. 2). *Escherichia coli* was also isolated in about 40 % of the cases independently of the clinical setting, but was far less common in proximal than in distal intestinal lesions. The opposite was true for *Candida* spp., being cultured in about 70 % of gastroduodenal lesions, but in less than 20 % of those in the colon or rectum.

**Table 1:** Case characteristics

	Community acquired	Postoperative	All
n (m/f)	88 (44/44)	154 (94/60)	242 (138/104)
Age^a^	70 (14–98)	65 (18–89)	66.5 (14–98)
Died in hospital (%)	17 (19.3 %)	28 (18.2 %)	45 (18.6 %)
LoS ICU^b^	2.5 (1–7.25)	9 (3–21)	5 (2–17)
LoS hospital^b^	12 (8–19.25)	23.5 (14–42)	18 (10–34)
Site of lesion			
Stomach/duodenum	13 (14.8 %)	10 (6.5 %)	23 (9.5 %)
Small intestine	15 (17.0 %)	33 (21.4 %)	48 (19.8 %)
Colon/rectum	37 (42.0 %)	47 (30.5 %)	84 (34.7 %)
Other or multiple	23 (26.1 %)	64 (41.6 %)	87 (36.0 %)
Isolated pathogens			
*E. coli*	40 (45.5 %)	63 (40.9 %)	103 (42.6 %)
Non-*E. coli* enterobacteriaceae	26 (29.5 %)	54 (35.1 %)	80 (33.1 %)
*Enterococcus* spp.	30 (34.1 %)	84 (54.5 %)	114 (47.1 %)
*Streptococcus* spp.	26 (29.5 %)	24 (15.6 %)	50 (20.7 %)
Anaerobes	32 (36.4 %)	40 (26.0 %)	72 (29.8 %)
*Candida* spp.	26 (29.5 %)	44 (28.6 %)	70 (28.9 %)
Non-fermenter	7 (8.0 %)	8 (5.2 %)	15 (6.2 %)
*S. aureus*	4 (4.5 %)	9^c^ (5.8 %)	13 (5.4 %)
CoNS	5 (5.7 %)	15 (9.7 %)	20 (8.3 %)
Others	8 (9.1 %)	11 (7.1 %)	19 (7.9 %)

^a^median (range)

^b^median (interquartile range)

^c^3 isolates with methicillin-resistance

**Table 2:** Pathogens cultured from intraabdominal swabs in 242 patients with secondary peritonitis

Escherichia coli (107)	Anaerobes (100) *Actinomyces turicensis* (1) *Bacteroides* *caccae* (2) *Bacteroides* *capillosus* (1) *Bacteroides* *eggerthii* (1) *Bacteroides* *fragilis* (33) *Bacteroides* *intestinalis* (1) *Bacteroides* *ovatus* (8) *Bacteroides* sp. (2) *Bacteroides* *stercoris* (1) *Bacteroides* *thetaiotaomicron* (7) *Bacteroides* *uniformis* (2) *Bacteroides* *vulgatus* (13) *Clostridium* *fallax* (1) *Clostridium* *perfringens* (1) *Clostridium* *septicum* (1) *Clostridium* *sordellii* (1) *Clostridium* sp. (1) *Clostridium* *symbiosum* (1) *Clostridium* *tertium* (2) *Fusobacterium* *necrophorum* (1) *Fusobacterium* *nucleatum* (2) *Fusobacterium* sp. (1) *Fusobacterium* *varium* (1) *Lactococcus* *garvieae* (1) *Leuconostoc* sp. (1) *Parabacteroides* *distasonis* (8) *Parabacteroides* *johnsonii* (1) *Pediococcus* *acidilactici* (1) *Peptostreptococcus* *anaerobius* (1) *Prevotella* *denticola* (2)	Streptococcus spp. (61) *S. agalactiae* (1) *S. anginosus* (19) *S. constellatus* (6) *S. cristatus* (1) *S. dysgalactiae* (4) *S. gordonii* (1) *S. infantarius* (2) *S. intermedius* (2) *S. massiliensis* (1) *S. mitis/oralis* (11) *S. parasanguinis* (3) *S. peroris* (1) *S. salivarius* (3) *S. sanguinis* (4) *S. vestibularis* (1) *Streptococcus* sp. (1)
Non-*E. coli* Enterobacteriaceae (105) *Citrobacter braakii* (3) *Citrobacter freundii* (9) *Enterobacter aerogenes* (1) *Enterobacter cloacae* (21) *Hafnia alvei* (3) *Klebsiella oxytoca* (13) *Klebsiella pneumoniae* (26) *Morganella morganii* (2) *Proteus mirabilis* (13) *Proteus penneri* (5) *Proteus vulgaris* (9)		
*Candida* spp. (77) *C. albicans* (33) *C. krusei* (1) *C. dubliensis* (1) *C. glabrata* (24) *C. krusei* (1) *C. lusitaniae* (1) *C. norvegensis* (1) *Candida* sp. (13) *C. tropicalis* (2)		Others (21) *Aerococcus viridans* (1) *Aeromonas veronii* (1) *Bacillus cereus* (1) *Bacillus licheniformis* (1) *Bifidobacterium* sp. (1) *Corynebacterium* sp. (1) *Corynebacterium* *striatum* (1) *Gemella* *morbillorum* (2) *Geotrichum* sp. (1) *Lactobacillus* *brevis* (1) *Lactobacillus* *fermentum* (1) *Lactobacillus* *gasseri* (1) *Lactobacillus* *paracasei* (1) *Lactobacillus* *paraplantarum* (1) *Lactobacillus* *rhamnosus* (1) *Lactobacillus* *salivarius* (1) *Lactobacillus* sp. (2) *Rothia* *mucilaginosa* (1) *Sphingomonas* *paucimobilis* (1)
*Staphylococcus aureus* (13) MSSA (10) MRSA (3)		
*Enterococcus* spp. (134) *E. avium* (11) *E. casseliflavus* (1) *E. dispar* (1) *E. faecalis* (61) *E. faecium* (56) *E. gallinarum* (3) *E. hirae* (1)	Non-fermenter (15) *Acinetobacter* *baumannii* (2) *Pseudomonas* *aeruginosa* (12) *Stenotrophomonas* *maltophilia* (1)	
	CoNS (21) *S.* *capitis* (2) *S. epidermidis* (14) *S. haemolyticus* (5)

The rates of susceptibility and resistance for the most relevant categories of pathogens are shown in Table 3. Generally, susceptibility rates were lower in postoperative cases. The same trend was observed for SAR (Table 4). Against enterobacteriaceae, CTX was superior to TZP when used alone (c.a./p.op. 95.5/83.8 vs. 86.4/69.5 %). Combinations of these two with GEN enhanced the respective SAR to 96.8–98.9 %. As for TGC, the SAR for monotherapy was only ~84 % against enterobacteriaceae due to the frequent isolation of resistant *Proteus* spp., along with some isolates of *K. pneumoniae* or *E. cloacae* with intermediate susceptibility or resistance. This could be amended by combination of TGC with CTX, CIP or GEN (SAR 92.2–99.4 %). Monotherapy of CIP would have been active in 87.7–93.2 % of patients, and MEM in 100 %.

**Table 3:** Rates of susceptibility and resistance in important categories of pathogens isolated in patients with secondary peritonitis

	*E. coli*			Non-*E. coli* enterobacteriaceae			*Enterococcus* spp.			*Candida* spp.		
	n	%S	%R	n	%S	%R	n	%S	%R	n	%S	%R
SAM												
c.a.	41	63.4	36.6	36	61.1	38.9	36	66.7	30.6			
p.op.	66	48.5	51.5	68	44.1	55.9	98	65.3	35.7			
TZP												
c.a.	41	78.0	9.8	36	91.7	2.8	^a^					
p.op.	66	60.6	19.7	68	61.8	23.5						
CXM												
c.a.	41	87.8	12.2	36	66.7	33.3						
p.op.	66	75.8	24.2	67	55.2	44.8						
CTX												
c.a.	41	95.1	0	36	94.4	5.6						
p.op.	66	84.8	6.1	69	72.5	23.2						
ETP												
c.a.	41	100	0	36	97.2	2.8						
p.op.	66	100	0	66	92.4	7.6						
MEM												
c.a.	41	100	0	36	100	0	^a^					
p.op.	66	100	0	69	100	0						
CIP												
c.a.	41	82.9	14.6	36	97.2	2.8						
p.op.	66	72.7	22.7	69	97.1	1.4						
GEN												
c.a.	41	97.6	2.4	36	97.2	2.8						
p.op.	66	92.4	7.6	69	97.1	2.9						
TGC												
c.a.	41	95.1	2.4	36	58.3	33.3	36	100	0			
p.op.	66	100	0	69	55.1	30.4	98	100	0			
VAN												
c.a.							36	94.4	5.6			
p.op.							97	88.7	11.3			
LZD												
c.a.							36	97.2	2.8			
p.op.							98	99.0	1.0			
FLC												
c.a.										21	76.2	4.8
p.op.										37	56.8	10.8

missing to 100 % intermediate susceptibility

*n* number of tested isolates,  *%S* susceptible,  *%R* resistant

^a^Susceptibility of *Enterococcus* spp. against TZP and MEM was assumed to be identical to that against SAM, which was inferred from that against ampicillin

**Table 4:** Spectrum adequacy of selected antimicrobial regimens in patients presenting with secondary peritonitis (88 community acquired cases, c.a.; 154 postoperative cases, p.op.)

	TZP		TZP + GEN		TZP + VAN		TZP + LZD	
	c.a.	p.op.	c.a.	p.op.	c.a.	p.op.	c.a.	p.op.
Enterobacteriaceae								
%S	86.4	69.5	97.7	96.8				
%R	5.7	18.2	0	0				
Enterobacteriaceae plus *enterococci*								
%S	72.7	58.4	84.1	75.3	85.2	67.5	85.2	69.5
%R	18.2	33.8	12.5	21.4	6.8	22.1	6.8	18.2

	CTX		CTX + GEN		CTX + VAN		CTX + LZD	
	c.a.	p.op.	c.a.	p.op.	c.a.	p.op.	c.a.	p.op.
Enterobacteriaceae								
%S	95.5	83.8	98.9	97.4				
%R	2.3	11.7	1.1	1.3				
Enterobacteriaceae plus *enterococci*								
%S	62.5	41.6	65.9	43.5	93.2	77.9	94.3	83.1
%R	35.2	57.1	34.1	55.2	4.5	16.9	3.4	12.3

	MEM				MEM + VAN		MEM + LZD	
	c.a.	p.op.	c.a.	p.op.	c.a.	p.op.	c.a.	p.op.
Enterobacteriaceae								
%S	100	100						
%R	0	0						
Enterobacteriaceae plus *enterococci*								
%S	87.5	78.6			98.9	94.2	98.9	99.4
%R	12.5	20.8			1.1	5.8	1.1	0.6

	CIP				CIP + VAN		CIP + LZD	
	c.a.	p.op.	c.a.	p.op.	c.a.	p.op.	c.a.	p.op.
Enterobacteriaceae								
%S	93.2	87.7						
%R	5.7	9.7						
Enterobacteriaceae plus *enterococci*								
%S	63.6	39.6			90.9	80.5	92.0	87.0
%R	12.5	37.0			5.7	13.0	5.7	10.4

	TGC		TGC + GEN		TGC + CIP		TGC + CTX	
	c.a.	p.op.	c.a.	p.op.	c.a.	p.op.	c.a.	p.op.
Enterobacteriaceae								
%S	84.1	83.1	97.7	99.4	98.9	98.7	98.9	92.2
%R	11.4	13.0	1.1	0.6	0	0.6	0	1.9
Enterobacteriaceae plus *enterococci*								
%S	84.1	83.1	97.7	99.4	98.9	98.7	98.9	92.2
%R	11.4	13.0	1.1	0.6	0	0.6	0	1.9

	FLC							
	c.a.	p.op.	c.a.	p.op.	c.a.	p.op.	c.a.	p.op.
*Candida* spp.								
%S	87.5	81.9						
%R	1.1	2.6						

%S: all pathogens of the respective categories are susceptible to the tested antimicrobial regimen, or no pathogen of the respective categories was isolated.  %R: at least one pathogen of the respective categories is resistant to all agents of the tested regimen. Missing to 100 %: no full, but intermediate or unknown susceptibility of at least one pathogen to at least one agent

When enterobacteriaceae and *enterococci* were evaluated together, the SAR of CTX-based regimens without anti-enterococcal activity dropped to 41.6–65.9 %. Due to the high occurrence of ampicillin-resistance in *enterococci* (~1/3), the SAR of TZP also decreased (58.1–84.1 %). Due to a percentage of 5.6–11.3 % of resistance to VAN among all isolated *enterococci* (mainly in *E. faecium*), these effects were only partially reversed by addition of VAN, but almost fully by addition of LZD. Since resistance to TGC was not observed among *enterococci*, SARs of its combinations were identical to those against enterobacteriaceae only. Assuming anti-enterococcal activity of MEM (the MICs of MEM for *enterococci* are typically two- to fourfold higher than for ampicillin or imipenem), the hit-rate of MEM was determined by the presence of ampicillin-resistant *enterococci*, and was 78.6–87.5 % when used as single agent, 94.2–98.9 % when combined with VAN and 98.9–99.4 % when combined with LZD.

Since antibacterial and antifungal agents do not share any common spectrum, the SAR of FLC was evaluated separately, and was 81.9–87.5 %. Of note, FLC-resistant strains were isolated only in 1.1–2.6 % of patients, whereas there was a considerable number of strains with intermediate (n = 16) or undetermined (n = 19) susceptibility.

## Discussion

With the present study, we provide an evaluation of antimicrobial agents, used alone or in combination, in a real-life data set of culture results from patients with secondary peritonitis who required intermediate or intensive care. It is important to stress the virtual character of this analysis; a regimen was considered adequate when all relevant pathogens isolated in one patient were susceptible to at least one of its components. No information on the clinical efficacy of the actual treatment was obtained or analysed. The advantage of this approach is that many potential regimens can be tested within a specific clinical and epidemiological context, and the results can be used e.g. to adjust local or regional treatment guidelines, or select promising candidate regimens for clinical trials. In contrast to ordinary surveillance data which report activity of single agents against single species (or genera), this approach also allows to evaluate the effect of the combination of more than one agent and more than one pathogen, as is usually the case in secondary peritonitis.

Representativeness of pathogen data is crucial. Although data quality (with regard to patient characteristics and diagnostic procedures) may be highest in clinical trials e.g. of new antimicrobial agents, such studies are likely to represent a highly selected population of tendentially less severe cases (particularly appendicitis) in younger and healthier patients than encountered in clinical practice [9, 10]. For the present study, the most important criterion for inclusion was admission to the surgical IMCU/ICU. Patients with clearly mild disease and good general health were therefore not included into this analysis, as evidenced by the high mortality of 18.5 %. The distribution of pathogens was generally similar to that reported by other studies [11–13]. Susceptibility rates were in fair agreement with the general surveillance data from our hospital. Carbapenemase-producing enterobacteriaceae (CRE) are still not endemic and were not observed in this study, whereas about 10 % of *E. coli* express ESBL, and about 25 % are resistant to fluoroquinolones. Resistance rate to vancomycin is found in about 20 % of *E. faecium*. For almost all agents, resistance rates were higher in postoperative than in community acquired cases, as has been described previously [13].

Anaerobes, *streptococci*, *staphylococci* and non-fermenters were excluded from the spectrum adequacy evaluation. There is consensus that activity against anaerobes should be provided in most cases [5, 14], and agents lacking such activity (e.g. CTX, CIP) are typically combined with metronidazole to close this gap. No drug-resistance at all was detected in *streptococci*, and their inclusion would therefore not change the results of the analysis. Coagulase-negative *staphylococci* are mostly resistant to betalactam antibiotics (18 out of 21 isolates in this study), but are considered rather contaminants than pathogens without the context of peritoneal dialysis. Some strains of *Staphylococcus aureus* and non-fermenters were isolated, both in community acquired (4 *S. aureus*, 6 *P. aeruginosa*, 1 *A. baumannii*) and postoperative cases (9 *S. aureus*, 6 *P. aeruginosa*, 1 *A. baumannii*, 1 *S. maltophilia*). Two isolates of P. aeruginosa isolated in postoperative cases were resistant to TZP and either ceftazidime or MEM, respectively, but fully susceptible to CIP and GEN. All other non-fermenters showed “wild type” susceptibility to all eligible agents. Only three isolates (all in postoperative cases) of *Staphylococcus aureus* were methicillin-resistant. Due to the low numbers of MRSA and non-fermenters, their impact on the present analysis was expected to be insignificant and of little reliability. This would probably be different if follow-up swabs of repeated laparotomies in cases with unfavourable evolution were considered, in which fastidious pathogens with multi-drug resistance may even dominate, and possibly be responsible for the insufficient response to therapy.

The main finding of this analysis is that even in an environment with moderate resistance rates among enterobacteriaceae, popular antimicrobial regimens such as TZP or CTX (plus metronidazole) provided only limited coverage (69.5–95.5 %) when all enterobacteriaceae found in one patient were considered. Combination of these agents with an aminoglycoside—as suggested by the IDSA guidelines [5] when resistant organisms are suspected—could considerably improve the spectrum adequacy rate. No carbapenem-resistant enterobacteriaceae were observed in this study, leaving carbapenems such as MEM as a theoretically fail-safe option.

When *enteroccoci* are of concern, the intrinsically active betalactams (TZP, MEM) alone did not provide reliable coverage due to a high percentage of ampicillin-resistant strains. This calls for combination with linezolid or vancomycin. Particularly with regard to *enterococci*, TGC offers an interesting spectrum, with no resistance observed in this study. Additionally, TGC has activity in multi-drug resistant enterobacteriaceae, including CRE, and it is approved as monotherapy for treatment of complicated intra-abdominal infection. However, the SAR of TGC monotherapy remained below 85 %. It is interesting that these rates were nearly identical in community acquired and postoperative cases, maybe indicating that the mechanisms leading to increasing resistance to other agents do not (yet) apply in the same way to TGC. In fact, reasons for inadequacy were mostly isolation of *Proteus* spp., which is generally easy to treat, but not a suitable target for TGC. Addition of CIP or GEN (or less effectively CTX) could close this gap. However, tigecycline has been associated with increased mortality and noncure-rates [15], and should be reserved for situations when alternative treatments are not suitable [16]. The rather low plasma concentrations compared to sensitivity breakpoints and the bacteriostatic activity of tigecycline have been discussed as potential explanations for the higher rate of treatment failure [15], but the defined limitations in spectrum (*Proteus* spp., *P. aeruginosa*) might also contribute. Although this is purely speculative, there may be more than one good reason for combination of tigecycline with a “classical” bactericidal agent such as a betalactam, a fluoroquinolone or an aminoglycoside.

Bearing the limitations of our analysis in mind, these results may offer some guidance on what antibacterial regimen to choose in cases of the highest severity and risk. Considering the regimens with a SAR of 95–100 %, these would be triple combinations of TZP or CTX (each plus GEN plus VAN or LZD), or double combinations with MEM (plus VAN or LZD) or TGC (plus CIP or GEN) for community acquired or postoperative peritonitis. For cases of lower severity and risk, regimens with lower SAR can be selected. Since the SAR is a patient-based index, differences can be directly translated into a *number needed to treat*. Irrespective of the absolute value, a regimen with a 5 % lower SAR will leave 1 additional patient out of 20 with formally inadequate spectrum, which may or may not affect clinical outcome. This consideration may be particularly helpful when the alternative with higher SAR is either costly (e.g. LZD, TGC) or toxic (e.g. GEN, VAN).

Some limitations of our analysis must be discussed. First, because all data were obtained retrospectively, several relevant aspects of the case characteristics were no longer accessible to us, particularly those on the individual antimicrobial treatment. All samples were probably obtained after some antibiotic (at least perioperative prophylaxis for laparotomy) had been administered. It is therefore possible that some susceptible pathogens may have been lost to detection. This would be problematic if ultra-broad spectrum antibiotics or combinations had often been used (eradicating even pathogens of “typical” resistance), or if a large number of samples had appeared to be completely sterile (excluding these cases completely from the analysis). Then, the prevalence of the more persistent and/or resistant pathogens could have been grossly overestimated. However, the microbiological patterns we observed are fairly consistent with results from other studies and our local surveillance data. Severe underrepresentation of highly susceptible pathogens (e.g. *streptococci*) would be of academic, but not of therapeutic importance. Second, antimicrobial susceptibility testing may be inaccurate. In particular, variable results have been reported for TZP with automated systems [17, 18]. The epidemiological cut-off values (ECOFF) of most enterobacteriaceae for TZP is identical (8 mg/L) to the susceptibility breakpoint (EUCAST rationale documents: http://www.eucast.org/documents/rd), and small variations of the test result may easily lead to categorisation of susceptible strains as intermediate. For cefotaxime, in contrast, the categorisation is probably more robust because the ECOFF is typically one or several log2-steps lower than the breakpoint. However, it is unlikely that erroneous categorisation of some isolates would have relevant effects on the global trends of our analysis. Third, our assessment of spectrum-adequacy was done irrespective of relative pathogenic relevance of the isolates, which is unlikely to represent the biological reality of secondary peritonitis. However, to our knowledge, these aspects are not sufficiently understood to be included into a model with clinical data; given the rather loose association between antimicrobial therapy and therapeutic success, there is certainly still much to be learned about the pathogen-host interaction in secondary peritonitis. All in all, although more detailed and refined analysis are certainly possible, we consider our results to be valid and clinically relevant.

## Conclusions

We developed a simple but innovative quantitative method to assess the spectrum adequacy of antimicrobial regimens to be used for polymicrobial infections (“battleship approach”), and applied it to a real-life data set of pathogens cultured in patients with secondary peritonitis admitted to the IMCU/ICU. The results of this approach can be used as a rational base to inform e.g. the local adaption of guidelines.

## Abbreviations

SAM: ampicillin/sulbactam
TZP: piperacillin/tazobactam
CXM: cefuroxime
CTX: cefotaxime
ETP: ertapenem
MEM: meropenem
CIP: ciprofloxacin
GEN: gentamicin
TGC: tigecycline
VAN: vancomycin
LZD: linezolid
FLC: fluconazole
